# Biomarker Research in NSAID Hypersensitivity: A Scoping Review and Evidence Map

**DOI:** 10.3390/ph19060838

**Published:** 2026-05-27

**Authors:** Yu Kyoung Hwang

**Affiliations:** Division of Allergy, Department of Internal Medicine, Chungbuk National University Hospital, Cheongju-si 28644, Republic of Korea; ashleyhwang11@gmail.com

**Keywords:** NSAID hypersensitivity, biomarker, scoping review, evidence mapping, NERD, aspirin-exacerbated respiratory disease, translational validation

## Abstract

**Background/Objectives**: Nonsteroidal anti-inflammatory drug (NSAID) hypersensitivity comprises clinically distinct phenotypes, with different implications for diagnosis and future drug use. However, the biomarker literature in this field remains heterogeneous and difficult to interpret as a whole. We aimed to map how NSAID hypersensitivity biomarker studies are distributed across harmonized phenotype categories, biomarker classes, intended uses, and translational validation stages. **Methods**: We conducted a scoping review with systematic literature searching and descriptive evidence mapping. English-language records published between 1 January 2005 and 31 March 2026 were identified through PubMed and Embase. After staged screening and cross-database deduplication, eligible records were classified according to prespecified phenotype, biomarker, intended use, reference-standard, and validation-stage frameworks. **Results**: A total of 218 deduplicated records were retained in the master dataset. The mapped literature was heavily concentrated in NSAID-exacerbated respiratory disease/aspirin-exacerbated respiratory disease (NERD/AERD) (189/218, 86.7%). Genetic biomarkers were the most frequent class (118/218, 54.1%). The most frequent phenotype–biomarker class connection was between NERD/AERD and genetic biomarkers (*n* = 100). Most records were mapped to phenotype/endotype stratification (152/218, 69.7%), and most remained at Stage 0 exploratory discovery (197/218, 90.4%). **Conclusions**: The NSAID hypersensitivity biomarker literature is concentrated in a narrow phenotype space and remains dominated by exploratory research with limited later-stage validation. Future studies should prioritize clearer phenotype harmonization, stronger diagnostic anchoring, standardized biospecimen strategies, and independent validation to improve clinical translation.

## 1. Introduction

NSAID hypersensitivity is a clinically important subset of adverse drug reactions that includes respiratory, cutaneous, and systemic phenotypes, each with distinct implications for diagnosis and future drug use [1]. NSAID may also act as clinically important cofactors in food-dependent exercise-induced anaphylaxis and related food-induced anaphylactic reactions [2,3,4]. In routine practice, diagnostic evaluation remains difficult because clinically recognized categories differ in cross-reactivity patterns, symptom profiles, and underlying biology [5,6]. Although drug provocation and other challenge-based approaches remain central in many settings, they are labor-intensive, not always feasible, and unsuitable for repeated or broad clinical application [6]. These limitations have sustained interest in biomarkers that support phenotypic classification, improve diagnostic precision, and reduce reliance on challenge-based testing.

A wide range of candidate biomarkers has been reported in NSAID hypersensitivity, including genetic, transcriptomic, proteomic, metabolomic, soluble, cellular, tissue-based, and multi-marker approaches [7,8]. However, this literature has not developed around a single translational objective. Some studies focus primarily on biological characterization, others on phenotype stratification, and a smaller proportion of diagnostic or prediction-oriented applications [9]. As a result, the overall translational structure of the field is difficult to understand from individual studies alone [10,11].

Another major obstacle is the heterogeneity of phenotype definition, clinical reference standards, and sampling context across studies. Investigators do not consistently distinguish among harmonized NSAID hypersensitivity categories, and many studies combine phenotypes or use incomplete diagnostic labels [12,13]. Reference standards also vary, ranging from challenge-based confirmation to history-based, mixed, or unclear diagnostic anchoring. At the same time, biomarkers have been assessed across diverse biospecimen sources and collection contexts, limiting direct comparison and weakening progress toward validation and clinical use [1,6,14,15].

We therefore conducted a scoping review with systematic literature searching and descriptive evidence mapping of biomarker studies in NSAID hypersensitivity. We mapped the literature according to harmonized phenotype categories, biomarker classes, intended uses, reference-standard relevance, and prespecified validation stages. Our aim was to identify where the evidence is concentrated, which clinically recognized phenotype groups remain underrepresented, and which structural features impede translation from candidate-marker discovery to clinically actionable use.

## 2. Materials and Methods

### 2.1. Study Design and Reporting Framework

We conducted a scoping review with systematic database searching and screening-level evidence mapping to characterize the distribution and translational orientation of biomarker research in NSAID hypersensitivity. A scoping approach was selected because the aim was to map the field, organize records across prespecified analytic domains, and identify areas of concentration and underrepresentation, rather than estimate pooled effect sizes [10,16]. The review was developed and reported with reference to the PRISMA extension for scoping reviews (PRISMA-ScR) [10,17]. A review protocol was not prospectively registered.

### 2.2. Review Questions

The primary review question was as follows: how was the literature on NSAID hypersensitivity biomarkers distributed across phenotype category, biomarker class, intended use, reference-standard relevance, and translational validation stage? Secondary questions addressed which phenotype categories were most represented, which biomarker classes were reported most often, what the main proposed uses of these biomarkers were, and how closely the literature was linked to reference-standard-oriented contexts or later-stage validation.

### 2.3. Eligibility Criteria

Records were considered eligible if they addressed NSAID hypersensitivity or closely related phenotype-specific entities and if the title or available bibliographic information indicated a biomarker-centered focus. Eligible records included studies on candidate biomarkers, biomarker profiles, molecular signatures, omics-related markers, diagnostic or predictive markers, and biomarker-based stratification approaches. Records were excluded if they were clearly unrelated to NSAID hypersensitivity, focused on aspirin or NSAID exposure in other clinical settings, addressed treatment efficacy or management without a biomarker-focused aim, represented reviews, editorials, guidelines, or other non-primary literature, were limited to nonhuman or in vitro-only work, were case reports or very small-scope reports, lacked sufficient abstract or bibliographic information for structured screening and mapping, or were identified as duplicates during consolidation.

### 2.4. Information Sources and Search Strategy

Systematic literature searches were conducted in PubMed and Embase. Separate database-specific strategies were developed using combinations of NSAID hypersensitivity phenotype terms and biomarker-related terms, with syntax adapted to each platform. The search window was restricted to publications from 1 January 2005 through 31 March 2026, and English-language records were targeted. The final searches were executed on 1 April 2026. The full operational search strategies for both databases are provided in Table S2. Because database content and indexing status change over time, retrieved record counts reflected database status on the search date.

### 2.5. Study Selection and Deduplication

Screening was carried out in stages within each database using a predefined decision framework. Records were initially assessed at the title/abstract or available bibliographic-information level. Excluded records were assigned predefined exclusion codes to preserve transparency in the screening process. After database-level screening, potentially relevant records were merged across sources and deduplicated across sources using title- and identifier-based matching, when available. The resulting deduplicated cross-database set was used as the master dataset for evidence mapping. All screening, structured annotation, classification, and evidence-mapping procedures were conducted by the single author.

### 2.6. Data Charting and Extraction Framework

A prespecified codebook was developed to support structured charting of the literature. For screened records, information was recorded in standardized fields covering screening decision, exclusion reason, phenotype clarity, biomarker centrality, candidate marker type, intended use, validation relevance, reference-standard relevance, and free-text notes. For records retained in the master dataset, these variables were used to support descriptive synthesis, figure construction, and table generation. Data charting and structured classification were performed by the single author using the predefined codebook framework.

### 2.7. Phenotype Classification Framework

To allow mapping across heterogeneous terminology, records were grouped into harmonized phenotype categories: NERD/AERD, NIUA, NECD, selective immediate reactions, selective delayed reactions, and mixed or phenotype-unclear records. This harmonization was based on predefined classification rules applied during structured screening and annotation. When the available screening information did not support confident assignment to a single phenotype category, the record was retained within the mixed/unclear category.

### 2.8. Biomarker Class and Intended-Use Framework

Records were further classified according to prespecified biomarker and intended-use domains. Biomarker classes included genetic, transcriptomic, proteomic, metabolomics/lipidomic, urinary mediator, serum/plasma marker, cellular assay, tissue marker, extracellular vesicle, and multimarker model categories. Intended-use categories included diagnostic replacement, diagnostic triage, phenotype/endotype stratification, mechanistic insight, severity or risk prediction, and treatment response or monitoring. These domains were applied to support descriptive mapping of the literature and to identify areas of concentration across the evidence base.

### 2.9. Reference-Standard Assessment

A separate reference-standard field was used to indicate whether a record appeared to be linked to challenge-based, history-based, mixed, unclear, or not-applicable diagnostic contexts. This variable was included because diagnostic anchoring was considered relevant to the translational interpretation of the biomarker literature. At the screening and mapping stage, reference-standard coding reflected structured annotation of the bibliographic record rather than formal adjudication of full-text diagnostic methodology.

### 2.10. Validation-Stage Framework

To position the mapped literature along a translational continuum, records were organized using a prespecified validation-stage framework [18,19]. This framework distinguished exploratory discovery-oriented studies, records suggestive of validation-oriented work, records linked to reference-standard-oriented clinical evaluation, and implementation-oriented contexts. The framework was used to support descriptive evidence mapping and should not be interpreted as a formal methodological or regulatory validation assessment [18,20]. Because screening-level information often did not allow for reliable separation of internal and external validation, stages 1 and 2 were grouped for descriptive mapping. Full-text review contributed selectively when additional clarification was required for phenotype assignment or validation-stage categorization. The operational rules used for validation-stage mapping were prespecified and are summarized in Table S1.

### 2.11. Evidence Mapping and Descriptive Analysis

After establishing the deduplicated master set, the literature was examined using descriptive evidence-mapping methods. Records were grouped according to phenotype category, biomarker class, intended use, and validation-related classification. These distributions were used to generate the principal outputs of the review: a phenotype-by-biomarker-class figure, an intended-use-by-validation-stage matrix, a summary characteristics table, and a conceptual bottleneck framework. Descriptive counts and proportions were used to characterize the structure of the literature and to identify areas in which the evidence base was concentrated or sparse.

## 3. Results

### 3.1. Search Results and Study Selection

**Figure 1:**
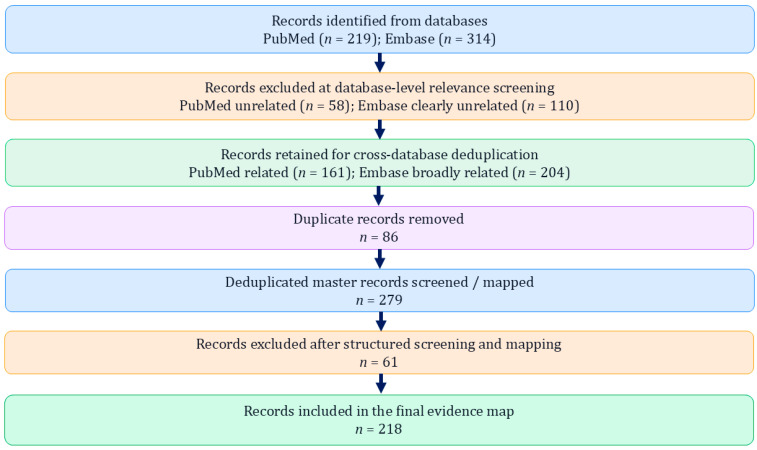
PRISMA flow diagram of record identification, staged screening, deduplication, and final inclusion. Records were identified from PubMed and Embase and underwent database-level relevance screening before cross-database deduplication. The retained records were then consolidated into a deduplicated master dataset and further screened using a structured evidence-mapping framework. A total of 218 records were included in the final evidence map.

A total of 533 records were initially identified from PubMed and Embase, including 219 from PubMed and 314 from Embase. After database-level relevance screening, 161 PubMed records and 204 Embase records were retained, yielding 365 records for cross-database deduplication. Removal of 86 duplicate records resulted in 279 deduplicated master records. Structured screening and mapping retained 218 records for the final evidence map (Figure 1). Records identified in a single database accounted for 139 of 218 records (63.8%), whereas those in both databases accounted for 79 of 218 records (36.2%). Embase contributed 160 of the 218 mapped records (73.4%), and PubMed contributed 137 (62.8%) (Table 1). Database-specific search approaches and screening rules are summarized in Tables S2 and S3. The full list of included records is provided in Table S4.

### 3.2. Distribution by Phenotype and Biomarker Class

The mapped literature was concentrated in the NERD/AERD phenotype category (Table 1). Of 218 included records, 189 (86.7%) were classified as NERD/AERD. NIUA, NECD, selective immediate, and mixed or phenotype-unclear categories accounted for only 10 (4.6%), 3 (1.4%), 6 (2.8%), and 10 (4.6%) records, respectively. No retained records were mapped to the selective delayed category. Across biomarker classes, genetic biomarkers were the most common class, accounting for 118 of 218 records (54.1%). Serum/plasma soluble markers accounted for 29 records (13.3%), followed by cellular/functional assays (17, 7.8%), metabolomics/lipidomic markers (13, 6.0%), tissue biomarkers (13, 6.0%), multi-marker models (10, 4.6%), urinary mediators (9, 4.1%), proteomic markers (6, 2.8%), transcriptomic markers (2, 0.9%), and extracellular vesicle biomarkers (1, 0.5%). The most frequent phenotype–biomarker class connection was between NERD/AERD and genetic biomarkers (*n* = 100) (Table 2). Other prominent links were NERD/AERD with serum/plasma markers (*n* = 27), tissue markers (*n* = 13), cellular assays (*n* = 11), and metabolomics/lipidomic markers (*n* = 11). Non-NERD/AERD phenotypes contributed only small numbers across biomarker classes. A supplementary connectivity visualization is provided in Figure S1.

### 3.3. Intended Use and Validation Stage

**Figure 2:**
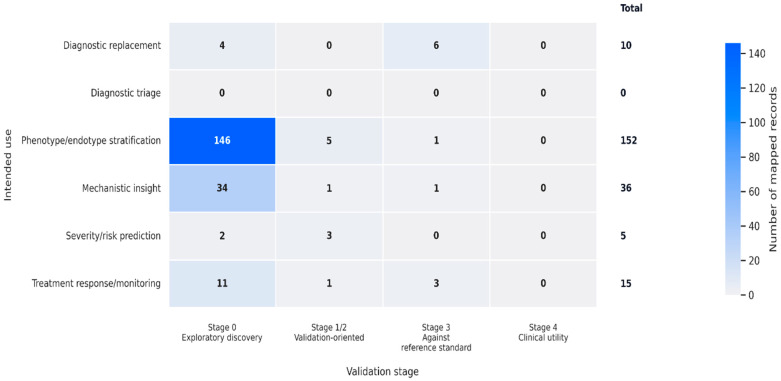
Distribution of mapped records across intended-use categories and prespecified translational validation stages. This heatmap summarizes the translational positioning of the mapped literature according to the primary intended use of each biomarker and its prespecified validation stage. Cell color intensity represents the number of mapped records. Most records clustered in exploratory discovery-oriented stages, whereas only a very small subset showed signals consistent with Stage 1/2 validation. A reference-standard-linked Stage 3 subset was present, but an implementation-oriented Stage 4 was absent. Operational definitions for validation-stage assignment are provided in Table S1.

Phenotype/endotype stratification was the most dominant intended-use category and accounted for 152 of 218 records (69.7%), followed by mechanistic insight (36/218, 16.5%) (Table 1). Diagnostic replacement (10/218, 4.6%) was less common. Validation-stage mapping showed that most records clustered in Stage 0 exploratory discovery (197/218, 90.4%), whereas only 10/218 records (4.6%) showed signals consistent with Stage 1/2 validation (Figure 2; Table 1). A Stage 3 reference-standard-linked subset was present (11/218, 5%). No records met the criteria for Stage 4 clinical utility. Table S1 summarizes the operational rules used for stage assignment.

### 3.4. Translational Bottlenecks and Readiness Signals

**Figure 3:**
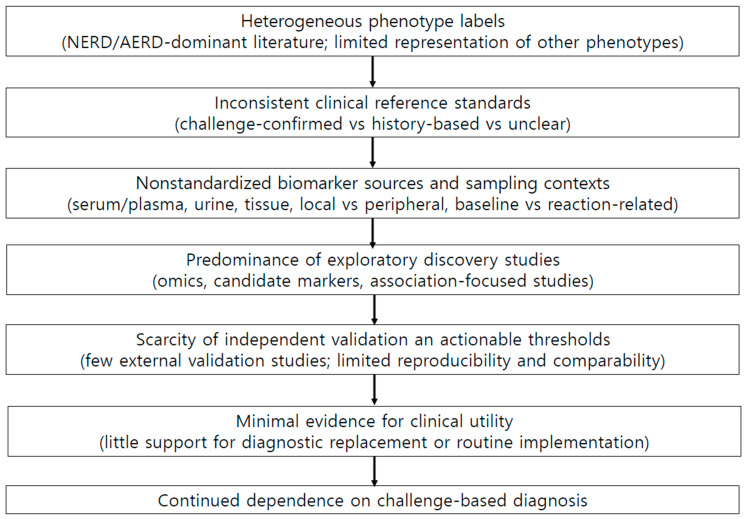
Conceptual summary of translational bottlenecks in NSAID hypersensitivity biomarker research. This conceptual diagram summarizes the principal barriers to clinical translation identified across the mapped literature in the master dataset, including fragmented phenotype definitions, inconsistent or weakly specified reference standards, nonstandardized biospecimen sources and sampling contexts, predominance of exploratory studies, limited independent validation, and scarcity of implementation-oriented evidence.

The mapped dataset showed a translational landscape characterized by concentration in a narrow phenotype space and limited progression beyond exploratory biomarker discovery. As summarized in Figure 3, the principal bottlenecks included fragmented phenotype definitions, inconsistent or weakly specified reference standards, heterogeneity in biospecimen source and sampling context, predominance of exploratory studies, limited independent validation, and absence of clear implementation-oriented evidence.

## 4. Discussion

This scoping review and evidence-mapping study showed three dominant patterns in the NSAID hypersensitivity biomarker literature. The evidence base was dominated by NERD/AERD, while other harmonized phenotypes were represented by only a small number of records. Genetic biomarkers formed the largest biomarker class, with serum/plasma soluble markers and cellular/functional assays appearing less frequently. Most records remained exploratory, with limited movement toward validation-oriented or implementation-ready evidence. Taken together, these findings indicate that the current literature contains a substantial number of candidate biomarker studies, but relatively little evidence has progressed to clinically actionable or implementation-stage use.

The concentration of studies in NERD/AERD phenotype contexts is clinically relevant because NSAID hypersensitivity comprises multiple distinct reaction types rather than a single disease entity [1]. NERD/AERD has become the dominant phenotype because it offers a relatively recognizable clinical and biological framework, including respiratory disease, eosinophilic inflammation, and mediator-driven pathology [14,21]. In contrast, other harmonized phenotype categories were represented by relatively few records. The absence of records mapped to selective delayed reactions may reflect the rarity and diagnostic ambiguity of this phenotype [6]. This imbalance limits the ability to assess whether candidate biomarkers are phenotype-specific and reduces comparability across studies [1,19].

The distribution of biomarker classes reflects the current state of the field. Genetic biomarkers were the most frequent class, followed by serum/plasma soluble markers and cellular/functional assays. This pattern indicates that the literature has generated a wide range of biological signals but has not converged on a small set of clinically stable markers [7,8,9]. Genetic, transcriptomic, and other discovery-oriented platforms were frequently represented, whereas mediator-based and cellular approaches were less commonly observed [8]. Consistent with this distribution, few biomarkers in the present map were supported by validation-oriented or implementation-stage evidence [18,19].

The intended-use and validation-stage distributions align with this pattern. Phenotype/endotype stratification and mechanistic insight accounted for most records, whereas diagnostic replacement and diagnostic triage were infrequent. Most studies were classified as Stage 0 exploratory discovery, with only a small proportion meeting criteria consistent with Stage 1/2 validation and no records identified at Stage 4 clinical utility. This absence likely reflects limited independent validation, inconsistent reference-standard linkage, and a lack of prospective clinical utility studies. A subset of studies was linked to reference standards (Stage 3), but this was not accompanied by evidence of reproducibility or implementation-stage evaluation. Overall, the mapped literature includes many candidate biomarkers but limited evidence supporting validated or clinically applicable use.

The bottlenecks summarized in Figure 3 help explain why this pattern persists. Phenotype definitions were not consistently harmonized across studies, and reference standards were variably specified, particularly with respect to challenge-based confirmation [1,6]. Biomarkers were also assessed across different biospecimen sources and sampling contexts, including blood, urine, tissue, and airway-related samples [6,9]. This heterogeneity makes direct comparison difficult and limits the accumulation of comparable evidence across studies. The problem is therefore not only that much of the literature remains exploratory, but also that exploratory work has often been conducted within a fragmented methodological framework.

This study has several strengths. It integrates phenotype classification, biomarker categories, intended use, and validation stage into a single mapping framework, allowing structural features of the literature to be examined in a unified way. By incorporating reference-standard relevance and translational positioning, the framework directly addresses the gap between biological plausibility and clinical applicability. This approach enables identification of where evidence is concentrated and where progression toward clinical use remains limited, which is difficult to assess from individual studies alone.

However, limitations should also be considered. As a scoping review with descriptive evidence mapping, it was designed to characterize distribution and translational structure rather than to estimate pooled biomarker performance. Some components of the mapping framework, including phenotype clarification and validation-stage assignment, were based on screening-level information and may not fully reflect details available in full-text methods. In addition, the mapped set depends on database indexing, search syntax, and codebook-based categorization. The English-language restriction may also have excluded relevant studies published in other languages. The literature search was limited to PubMed and Embase, which may have reduced coverage of records indexed in other databases. In addition, the review protocol was not prospectively registered. Because screening and classification were conducted by a single reviewer, the possibility of selection bias or classification bias cannot be excluded. This study does not address causality, but it describes how the literature is structured and the extent to which biomarker research has approached clinical translation.

## 5. Conclusions

This evidence map shows that NSAID hypersensitivity biomarker research is concentrated in a limited phenotype space and remains largely at the exploratory stage, with few studies reaching validation or implementation. This gap is clinically relevant because diagnosis still relies on challenge-based assessment, which carries procedural burden and uncertainty. As a scoping review based on descriptive mapping, this study does not assess biomarker performance or causality. Future work should focus on explicit reference-standard anchoring, more standardized biospecimen strategies, and independent validation to enable clinically interpretable use.

## Figures and Tables

**Table 1:** Characteristics of the mapped NSAID hypersensitivity biomarker literature in the deduplicated master dataset.

Section	Characteristic	*n*	%
Overall	Deduplicated unique included records	218	100
Database presence	PubMed	137	62.8
	Embase	160	73.4
Source overlap	Present in 1 database	139	63.8
	Present in 2 databases	79	36.2
Phenotype category	NERD/AERD	189	86.7
	NIUA	10	4.6
	NECD	3	1.4
	Selective immediate	6	2.8
	Mixed or phenotype unclear	10	4.6
	Selective delayed	0	0
Biomarker class	Genetic	118	54.1
	Transcriptomic	2	0.9
	Proteomic	6	2.8
	Metabolomic/lipidomic	13	6
	Urinary mediator	9	4.1
	Serum/plasma soluble marker	29	13.3
	Cellular/functional assay	17	7.8
	Tissue biomarker	13	6
	Extracellular vesicle	1	0.5
	Multimarker model	10	4.6
Intended use	Diagnostic replacement	10	4.6
	Diagnostic triage	0	0
	Phenotype/endotype stratification	152	69.7
	Mechanistic insight	36	16.5
	Severity/risk prediction	5	2.3
	Treatment response/monitoring	15	6.9
Validation axis	Stage 0: Exploratory discovery	197	90.4
	Stage 1/2: Validation-oriented	10	4.6
	Stage 3: Against reference standard	11	5
	Stage 4: Clinical utility	0	0

Abbreviations: AERD, aspirin-exacerbated respiratory disease; NERD, NSAID-exacerbated respiratory disease; NIUA, NSAID-induced urticaria/angioedema; NECD, NSAID-exacerbated cutaneous disease. Percentages were calculated using the unique master dataset as the denominator and are presented for descriptive evidence-mapping purposes.

**Table 2:** Major phenotype–biomarker class connections in the mapped literature.

Phenotype Category	Biomarker Class	Mapped Records (*n*)
Mixed/unclear	Genetic	5
	Cellular/functional assay	4
	Serum/plasma soluble marker	1
NECD	Cellular/functional assay	2
	Genetic	1
NERD/AERD	Genetic	100
	Serum/plasma soluble marker	27
	Tissue biomarker	13
	Cellular/functional assay	11
	Metabolomic/Lipidomic	11
	Multimarker model	10
	Urinary mediator	9
	Proteomic	6
	Extracellular vesicle	1
	Transcriptomic	1
NIUA	Genetic	8
	Metabolomic/Lipidomic	1
	Transcriptomic	1
Selective immediate	Genetic	4
	Metabolomic/Lipidomic	1
	Serum/plasma soluble marker	1

## Data Availability

No new data were created or analyzed in this study. Data sharing is not applicable to this article.

## Supplementary Materials

The following supporting information can be downloaded at https://www.mdpi.com/article/10.3390/ph19060838/s1, Figure S1: Phenotype concentration and phenotype-biomarker class connectivity in the mapped NSAID hypersensitivity literature; Table S1: Operational rules used for validation-stage mapping; Table S2: Database-specific search strategies used for literature identification; Table S3: Screening codebook and exclusion codes used for structured evidence mapping; Table S4: List of included records in the final evidence map.
