# Supplementary material for: Biomarker Research in NSAID Hypersensitivity: A Scoping Review and Evidence Map
Source: Pharmaceuticals (Basel). 2026 May 27;19(6):838. doi: 10.3390/ph19060838 (PMC13305205; doi:10.3390/ph19060838)
Supplement: Supplementary file 1 [file pharmaceuticals-19-00838-s001.zip › Supplementary Figure S1 and Tables S1-S3.pdf]

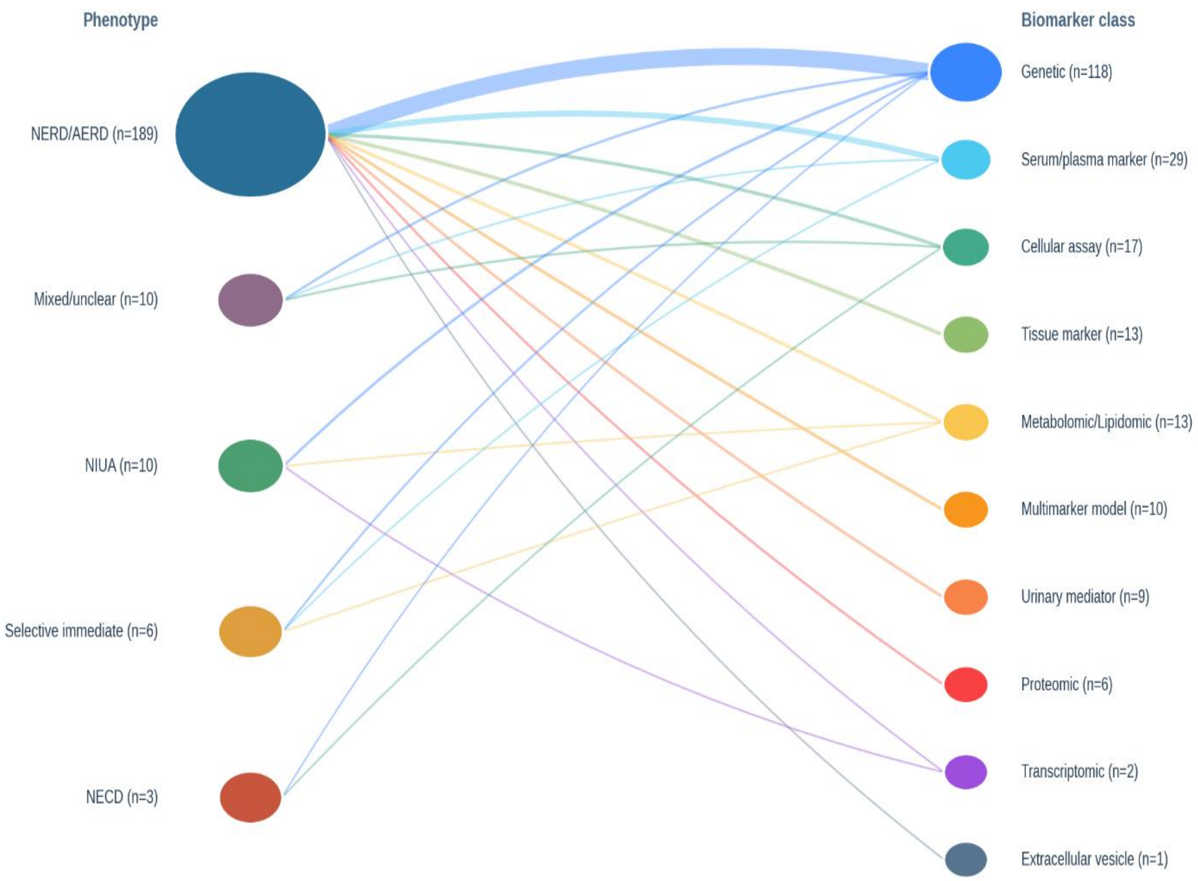

Figure S1. Phenotype concentration and phenotype–biomarker class connectivity in the mapped NSAID hypersensitivity literature.

This network-style visualization summarizes the distribution of mapped records across phenotype categories and their links to biomarker classes in the deduplicated master dataset. Node size reflects the total number of mapped records within each phenotype or biomarker category, and line width reflects the number of mapped records linking each phenotype-biomarker class combination. The figure highlights the marked concentration of the literature in NERD/AERD-related records, with particularly strong representation in genetic biomarkers and smaller but visible links to serum/plasma soluble markers, tissue biomarkers, metabolomics/lipidomic markers, and cellular/functional assays. Non-NERD phenotypes were represented by substantially fewer records and a narrower biomarker spectrum. Abbreviations: AERD, aspirin-exacerbated respiratory disease; NERD, NSAID-exacerbated respiratory disease.

Table S1. Operational rules used for validation-stage mapping

Validation stage	Operational definition used in this study	Typical cues used during mapping	Notes
**Stage 0: Exploratory discovery**	Records focused primarily on candidate biomarker discovery, biological association, omics profiling, mediator characterization, or subgroup description without clear evidence of validation or reference-standard-based performance assessment.	biomarker discovery, profile, signature, association, omics, mechanistic biomarker framing	Default category when no stronger validation signal was identifiable from screening-level data.
**Stage 1/2: Validation-oriented**	Records suggesting internal confirmation, model development and validation, derivation/replication, or external validation-oriented framing, but without clear evidence of routine clinical implementation.	validation, validated, prediction model, machine learning model development and validation, replication	In the present mapping, stage 1 and stage 2 were grouped because screening-level information was often insufficient to separate internal from independent external validation reliably.
**Stage 3: Reference-standard-linked clinical evaluation**	Records linked to diagnostic performance or evaluation against an explicit or likely reference-standard-oriented context, especially challenge-	challenge, provocation, diagnostic performance, sensitivity, specificity, aspirin	Stage 3 indicates clinically anchored evaluation, not necessarily independent validation.

	based or provocation-related settings.	challenge, drug provocation	
**Stage 4: Clinical utility / implementation**	Records explicitly addressing implementation, clinical utility, routine diagnostic deployment, or practice-oriented use beyond discovery/validation.	clinical utility, implementation, routine use, practice integration	No clear records were identified in this category in the draft mapping.

Validation-stage categories were assigned using prespecified operational rules for descriptive evidence mapping. Where screening-level information was insufficient to separate intermediate validation stages with certainty, records were grouped conservatively for mapping purposes.

Table S2. Database-specific search strategies used for literature identification

A. Search sources and coverage

Database	Platform / source	Date range	Language restriction	Search field approach	Notes
**PubMed**	MEDLINE via PubMed	1 January 2005 to 31 March 2026	English	Title/Abstract-based phenotype and biomarker terms with date filtering and human restriction	Search strategy combined NSAID hypersensitivity phenotype-related terms with biomarker-related terms.
**Embase**	Embase	1 January 2005 to 31 March 2026	English	Database-specific free-text strategy using phenotype-related and biomarker-related terms	Search syntax was adapted to the Embase interface and refined to reduce non-NSAID drug hypersensitivity noise.

B. Full operational search strings

**PubMed**	(<br>(<br>"aspirin-exacerbated respiratory disease"[tiab]<br>OR "NSAID-exacerbated respiratory disease"[tiab]<br>OR "aspirin-induced asthma"[tiab]<br>OR "aspirin-sensitive asthma"[tiab]<br>OR "aspirin-intolerant asthma"[tiab]<br>OR "aspirin hypersensitivity"[tiab]<br>OR "aspirin intolerance"[tiab]<br>OR "NSAID hypersensitivity"[tiab]<br>OR "nonsteroidal anti-inflammatory drug hypersensitivity"[tiab]<br>OR "non-steroidal anti-inflammatory drug hypersensitivity"[tiab]

	OR "NSAID-exacerbated cutaneous disease"[tiab]<br>OR "NSAID-induced urticaria/angioedema"[tiab]<br>OR "single-NSAID-induced urticaria/angioedema or anaphylaxis"[tiab]<br>OR "single-NSAID-induced delayed reactions"[tiab]<br>OR AERD[tiab]<br>OR NIUA[tiab]<br>OR NECD[tiab]<br>OR SNIUAA[tiab]<br>OR SNIDR[tiab]<br>OR "Samter triad"[tiab]<br>OR "Widal triad"[tiab]<br>)<br>AND<br>(<br>biomarker*[tiab]<br>OR genetic*[tiab]<br>OR genomic*[tiab]<br>OR transcriptomic*[tiab]<br>OR proteomic*[tiab]<br>OR metabolomic*[tiab]<br>OR lipidomic*[tiab]<br>OR polymorphism*[tiab]<br>OR variant*[tiab]<br>OR mutation*[tiab]<br>OR "gene expression"[tiab]<br>OR periostin[tiab]<br>OR leukotriene*[tiab]<br>OR LTE4[tiab]<br>OR prostaglandin*[tiab]<br>OR "15-HETE"[tiab]<br>OR "15-oxo-ETE"[tiab]<br>OR tryptase[tiab]<br>OR "basophil activation test"[tiab]<br>OR "eosinophil-derived neurotoxin"[tiab]<br>OR "fractional exhaled nitric oxide"[tiab]<br>OR "nitric oxide"[tiab]<br>OR FeNO[tiab]<br>OR exosome*[tiab]<br>OR "extracellular vesicle*"[tiab]<br>OR "EP2 receptor"[tiab]<br>OR "thromboxane receptor"[tiab]<br>)<br>)<br>AND english[lang]<br>AND ("2005/01/01"[dp] : "2026/03/31"[dp])<br>NOT (animals[mh] NOT humans[mh])<br>NOT (review[pt] OR editorial[pt] OR comment[pt] OR letter[pt])<br>NOT (<br>guideline[Title]<br>OR statement[Title]<br>OR "position paper"[Title]<br>OR review[Title]<br>OR update[Title]<br>OR management[Title]<br>OR preeclampsia[tiab]<br>OR pregnancy[tiab]<br>OR "eosinophilic asthma"[tiab]<br>OR "drug-induced anaphylaxis"[Title]<br>OR "world allergy organization"[Title]<br>OR "non-erosive reflux disease"[tiab]<br>OR GERD[tiab]<br>OR reflux[Title]<br>)

**Embase**	#1 phenotype<br>'aspirin-exacerbated respiratory disease':ti,ab OR 'nsaid-exacerbated respiratory disease':ti,ab OR 'aspirin-induced asthma':ti,ab OR 'aspirin-sensitive asthma':ti,ab OR 'aspirin-intolerant asthma':ti,ab OR 'aspirin hypersensitivity':ti OR 'aspirin intolerance':ti,ab OR 'nsaid hypersensitivity':ti OR 'nonsteroidal anti-inflammatory drug hypersensitivity':ti OR 'non-steroidal anti-inflammatory drug hypersensitivity':ti OR 'nsaid-exacerbated cutaneous disease':ti,ab OR 'nsaid-induced urticaria/angioedema':ti,ab OR 'single-nsaid-induced urticaria/angioedema or anaphylaxis':ti,ab OR 'single-nsaid-induced delayed reactions':ti,ab OR aerd:ti,ab OR 'n-erd':ti,ab OR niua:ti,ab OR sniuaa:ti,ab OR snidr:ti,ab OR 'samter triad':ti,ab OR 'widal triad':ti,ab OR 'widal syndrome':ti,ab OR 'aspirin-intolerant urticaria':ti,ab OR 'aspirin intolerant urticaria':ti,ab<br><br>#2 biomarker<br>biomarker*:ti,ab OR genetic*:ti,ab OR genomic*:ti,ab OR transcriptom*:ti,ab OR proteom*:ti,ab OR metabolom*:ti,ab OR lipidom*:ti,ab OR polymorphism*:ti,ab OR variant*:ti,ab OR mutation*:ti,ab OR 'gene expression':ti,ab OR methylation:ti,ab OR 'dna methylation':ti,ab OR eosinophil*:ti,ab OR periostin:ti,ab OR tryptase:ti,ab OR 'basophil activation test':ti,ab OR 'platelet-adherent basophils':ti,ab OR leukotriene*:ti,ab OR lte4:ti,ab OR prostaglandin*:ti,ab OR pgd2:ti,ab OR '15-hete':ti,ab OR '15-oxo-ete':ti,ab OR lipoxin*:ti,ab OR 'thromboxane receptor':ti,ab OR tbxa2r:ti,ab OR ptgdr2:ti,ab OR 'nitric oxide':ti,ab OR feno:ti,ab OR 'fractional exhaled nitric oxide':ti,ab OR 'eosinophil-derived neurotoxin':ti,ab OR exosome*:ti,ab OR 'extracellular vesicle':ti,ab OR 'extracellular vesicles':ti,ab OR 'endocannabinoid receptor':ti,ab OR cb2r:ti,ab OR ifetroban:ti,ab OR 'antibody-secreting cells':ti,ab OR 'proliferation signature':ti,ab<br><br>#3 exclusion<br>guideline:ti OR statement:ti OR 'position paper':ti OR review:ti OR update:ti OR management:ti OR 'natural history':ti,ab OR survey:ti,ab OR knowledge:ti,ab OR practice*:ti,ab OR 'healthcare worker*':ti,ab OR 'quality of life':ti,ab OR 'revision surgery':ti,ab OR 'endoscopic sinus surgery':ti,ab OR 'frontal sinus surgery':ti,ab OR sinusotomy:ti,ab OR postoperative:ti,ab OR postsurgical:ti,ab OR 'post-surgical':ti,ab OR biologic*:ti,ab OR tezepelumab:ti,ab OR mepolizumab:ti,ab OR dupilumab:ti,ab OR omalizumab:ti,ab OR benralizumab:ti,ab OR atad:ti,ab OR desensiti*:ti,ab OR 'aspirin tolerance':ti,ab OR 'central compartment atopic disease':ti,ab OR psoriasis:ti,ab OR 'non-erosive reflux disease':ti,ab OR gerd:ti,ab OR reflux:ti OR acetaminophen:ti,ab OR paracetamol:ti,ab OR 'necrotizing enterocolitis':ti,ab OR clopidogrel:ti,ab OR indobufen:ti,ab OR 'acute myocardial infarction':ti,ab OR covid*:ti,ab OR pregnancy:ti,ab OR preeclampsia:ti,ab<br><br>#4 final<br>#1 AND #2 NOT #3

Database-specific search strings were adapted to the syntax requirements of each platform. Searches were designed to identify biomarker-related literature in NSAID hypersensitivity and related phenotype-specific contexts. Retrieved records were subsequently screened, coded, merged across sources, and deduplicated before descriptive evidence mapping. Retrieved counts reflect database status on the search date and may differ on rerun.

Table S3. Screening codebook and exclusion codes used for structured evidence mapping

A. Screening decision categories

Variable	Category	Operational meaning
**screen_decision**	**include**	Record was considered sufficiently relevant to NSAID hypersensitivity biomarker mapping based on screening-level information.
	**maybe**	Record appeared potentially relevant but lacked sufficient clarity at screening level regarding phenotype specificity, biomarker centrality, or translational framing. Records coded as “maybe” were retained for further review and were subsequently adjudicated before final evidence mapping.
	**exclude**	Record did not meet the operational screening criteria for the mapped evidence base.

B. Core screening variables

Variable	Allowed values	Operational rule used in this study
**phenotype_clear**	yes / partial / no	“Yes” indicated that the title or screening-level information clearly supported an NSAID hypersensitivity phenotype or closely related phenotype-specific category. “Partial” indicated possible relevance but incomplete phenotype specificity. “No” indicated insufficient phenotype relevance.
**biomarker_central_question**	yes / partial / no	“Yes” indicated that a biomarker, molecular marker, profile, signature, or biomarker-oriented model appeared central to the record. “Partial” indicated that biomarker relevance was present but not clearly primary. “No” indicated that the record was not biomarker-focused.

**candidate_marker_type**	genetic; transcriptomic; proteomic; metabolomic_lipidomic; urinary_mediator; serum_plasma_marker; cellular_assay; tissue_marker; extracellular_vesicle; multimarker_model; other; none	Assigned according to the dominant biomarker modality suggested by the screening-level record.
**intended_use_prelim**	diagnostic_replacement; diagnostic_triage; phenotype_endotype_stratification; mechanistic_insight; severity_risk_prediction; treatment_response_monitoring; unclear; none	Assigned according to the apparent main translational or scientific purpose of the biomarker.
**validation_relevant**	yes / no / unclear	Used to indicate whether the record appeared to contain validation-oriented framing or clinically evaluable biomarker positioning.
**reference_standard_relevant**	challenge_based; history_based; mixed; unclear; not_applicable	Used to indicate whether the record appeared linked to challenge-oriented, history-based, mixed, or non-applicable diagnostic contexts.

C. Exclusion code system

Exclusion code	Label	Operational meaning
**E1**	not_NSAID_hypersensitivity	Record was not sufficiently related to NSAID hypersensitivity.
**E2**	aspirin_other_context	Record involved aspirin or NSAID exposure in a different clinical context unrelated to NSAID hypersensitivity biomarker mapping.
**E3**	not_biomarker_focused	Record was relevant to the disease area but did not have a biomarker-centered question.
**E4**	treatment_efficacy_only	Record focused mainly on treatment efficacy, intervention response, or management rather than biomarker mapping.
**E5**	mechanism_only	Record focused on pathogenesis or biological mechanisms without sufficient biomarker-centered framing.
**E6**	review_editorial_guideline	Record was a review, editorial, guidance, or similar non-target publication type for the mapped analysis.
**E7**	phenotype_unclear	NSAID hypersensitivity phenotype relevance was too unclear for inclusion in the mapped set.
**E8**	nonhuman_or_invitro_only	Record was limited to nonhuman or in vitro work without direct human biomarker mapping relevance.
**E9**	case_report_or_too_small	Record was a case report or too limited in scope for the mapped evidence base.
**E10**	no_relevant_abstract_info	Screening-level information was insufficient to support mapping relevance.
**E11**	duplicate	Record was identified as a duplicate during within-database or cross-database consolidation and removed during deduplication rather than substantive eligibility screening.
**E12**	other	Record was excluded for another reason not captured above.
